# Changes in attitudes toward psychothertionapy from medical school through residency graduation

**DOI:** 10.1192/j.eurpsy.2026.11727

**Published:** 2026-07-31

**Authors:** L. Mehl-Madrona, B. J. Mainguy

**Affiliations:** 1 Research; 2 Education, Coyote Institute, Orono, United States

## Abstract

**Introduction:**

Medical students applying for psychiatry residencies generally express strong interest in psychotherapy, seeing it as integral to their future roles. Surveys and systematic reviews consistently show that the majority of psychiatry residency applicants (and trainees) support the inclusion of psychotherapy in training, with 67–92% viewing being a psychotherapist as key to their psychiatric identity and around 88–98% supporting psychotherapy as an essential part of residency curricula. Despite strong initial interest, many residents report dissatisfaction with the quality of psychotherapy training they receive. Common concerns include inadequate supervision, limited clinical time, resource shortages, and financial constraints. These obstacles often lead to declining interest in psychotherapy over the course of residency, with senior residents less likely than juniors to plan to practice psychotherapy extensively.

**Objectives:**

We wanted to track changes in attitudes toward psychotherapy across the course of training.

**Methods:**

We interviewed 76 medical students applying for psychiatry residency and again during their residency, asking about attitudes toward psychotherapy and about their comfort with doing psychotherapy.

**Results:**

Consistent with prior findings, 84% of medical students expressed an interest in doing psychotherapy. However, only 52% of applicants could identify a form of psychotherapy that interested them. Pursuing further questions in humanities, only 18% could identify a philosopher whom they enjoyed. Forty-five percent could provide a definition of psychotherapy. During residency, the trainees reported on average being able to do 1.4 hours per week of psychotherapy during years 2 -4. Eighty-four percent felt uncomfortable with psychotherapy and preferred prescribing medications, an activity for which they felt they had expertise. In examining job prospects, of 49 available postings, none provided the opportunity to do psychotherapy.

**Conclusions:**

While an interest in psychotherapy is common among medical students, their understanding of the field is limited, and it diminishes quickly during training. Job opportunities that include psychotherapy are non-existent. In the United States, we speculate that economic forces are at work. Prescribing medications generates more income than doing psychotherapy alongside prescribing medications. Impetus exists to consider within the field of psychiatry, the role of prescribing alone versus prescribing and doing psychotherapy together.

**Disclosure of Interest:**

None Declared

